# Analysis of the Initial Learning Curve for Robotic-Assisted Total Knee Arthroplasty Using the ROSA^®^ Knee System

**DOI:** 10.3390/jcm13113349

**Published:** 2024-06-06

**Authors:** Inmaculada Neira, Rafael Llopis, Luis Cuadrado, David Fernández, Enrique Villanueva, Néstor Nuño, Francisco Forriol

**Affiliations:** 1 Department of Orthopedic Surgery, Hospital Universitario Santa Cristina, 28006 Madrid, Spain; rafa@drllopis.com (R.L.); cuadradorubioluis@gmail.com (L.C.); davidfr91@gmail.com (D.F.); dr.enriquevillanueva@gmail.com (E.V.); 2 Facultad de Medicina, Universidad CEU San Pablo, 28925 Alcorcón, Spain; fforriol@mac.com; 3 Orthopedic Surgery, Hospital General Tierra y Libertad, Monterrey 64325, Mexico; 4 Independent Researcher, 28400 Madrid, Spain; nuno7@hotmail.com

**Keywords:** total knee arthroplasty (TKA), robotic-assisted system, robotic-assisted TKA, ROSA Knee System, learning curve

## Abstract

**Background/Objectives:** Total knee arthroplasty (TKA) is a frequent procedure in orthopedic surgery. Advances in TKA include the development of robotic-assisted systems. Training in raTKA entails a learning curve to achieve proficiency comparable to conventional manual TKA (maTKA). **Methods:** We conducted a prospective study of the learning curve in raTKA using the Robotic Surgical Assistant (ROSA) Knee System. The study included 180 patients (90 raTKAs; 90 maTKAs) and three surgeons (one with >15 years of experience in maTKA). The cumulative sum control chart method (CUSUM) was used to define the transition from the learning phase to the mastered phase in raTKA. **Results:** The learning curves were 43 cases (experienced surgeons) and 61 cases (all surgeons). Mean operative times for both phases in raTKA were longer than in maTKA (*p* < 0.001). In raTKA, operative times in the learning phase were longer compared to those in the mastered phase (*p* < 0.001). Operative times in the learning and mastered phases for all surgeons in raTKA were significantly longer compared to those in maTKA (*p* < 0.001); however, operative times of the experienced surgeon in the mastered phase of raTKA and in maTKA showed no differences. **Conclusions:** The learning curve in raTKA is dependent upon the surgeon’s previous experience in maTKA.

## 1. Introduction

Total knee arthroplasty (TKA) for the treatment of symptomatic end-stage osteoarthritis is one of the most frequent procedures in orthopedic surgery. Patients undergoing TKA report overall satisfaction rates that range from 82% to 89% [1], but up to 20% develop pain, instability, and decreased range of motion [2,3,4]. Innovations introduced in the last two decades to reduce failure rates and increase patient satisfaction following TKA include navigation and robotic-assisted technologies [4,5,6,7,8]. By providing improved precision and accuracy in component positioning, robotic-assisted TKA (raTKA) might improve knee kinematics, expedite recovery, and improve the long-term survivorship of the implants [9].

Robotic-assisted systems are classified as passive, semi-active, and active [1]. Passive robotic systems provide positional guidance to the surgeon using computer-assisted or navigation technology. In semi-active systems, the surgeon performs bone resections under the guidance of a robotic arm and receives real-time feedback that limits deviations from presurgical planning. Active robotic systems operate autonomously, performing bone resections independently. Whereas passive robotic systems have limited use in TKA, both semi-active and active systems are increasingly being employed. Robotic-assisted systems can also be classified as image-dependent or imageless. In image-dependent systems, the surgical plan is based on a virtual three-dimensional (3D) model of the patient-specific bone anatomy derived from preoperative imaging. In contrast, imageless systems rely on intraoperative post-arthrotomy recordings of bony landmarks [1].

The ROSA (Robotic Surgical Assistant) Knee System (Zimmer-Biomet, Warsaw, IN, USA) is a semi-active robotic system for TKA that was launched in 2018 and was approved by the US Food and Drug Administration in January 2019. The ROSA Knee System can be considered collaborative robotics since the surgeon remains in control during the procedure while supported by an intelligent robotic tool to perform the surgery with high precision and reproducibility. The ROSA Knee System has two options for case creation and surgical plan development [10]. One option uses a virtual 3D model developed from preoperative plain X-ray studies using image processing software (version 1.2) and surface landmarks registered intraoperatively by the surgeon. The second option does not require preoperative images and is based exclusively on intraoperative post-arthrotomy landmark acquisition. The robotic arm assists the surgeon with the distal femoral cut, the sizing and positioning of the femoral component, the tibial cut, and ligament balance [11]. The surgeon operates the bone saw while being assisted by the robotic arm that holds the guide in the intended locations for the bone cuts.

As with most new surgical techniques, there is an associated learning curve for raTKA until surgeons achieve proficiency comparable to conventional TKA (maTKA). The ROSA Knee System became available in Spain in December 2020 and at our institution in January 2021. We present the results of a prospective study that analyzed the learning curve for the ROSA Knee System after it became available at our institution. The cumulative sum control chart method (CUSUM) was used for analysis [12]. In this method, the transition from the learning phase (until experience is obtained) to the post-learning phase (mastered phase) of a technique is indicated by the inflection point in the CUSUM curve. We found that operative times for both the learning and mastered phases in raTKA were longer than those in maTKA. Additionally, operative times in the learning phase were significantly longer compared to those in the mastered phase in raTKA. However, operative times of the experienced surgeon in the mastered phase of raTKA and in maTKA showed no differences. We conclude that the learning curve for raTKA is dependent upon the surgeon´s previous experience in maTKA.

## 2. Materials and Methods

### 2.1. Study Design

This prospective study was conducted at a single institution (Hospital Universitario Santa Cristina, Madrid, Spain) performing > 450 maTKAs per year to analyze the learning curve for raTKA using the ROSA Knee System. The study included adult patients with symptomatic tricompartmental or femur–tibial osteoarthritis who had an indication for TKA. Exclusion criteria were (i) previous knee surgeries or ipsilateral hip pathologies (operated or not), (ii) anatomical alterations like ligamentous instability or significant changes in the alignment between the femur and tibia, (iii) active infections near the knee joint, and (iv) a body mass index > 30. The study was conducted between February 2021 and June 2023 and included 180 patients: 90 underwent raTKA, and 90 underwent maTKA. Three orthopedic surgeons participated in the study: surgeon no. 1 had >15 years of experience in maTKA; surgeons no. 2 and no. 3 had <5 years of experience in maTKA. None of the surgeons had previous experience in navigation-assisted or robotic-assisted surgery. Surgeon no. 1 performed all maTKAs and participated in all raTKAs included in the study. The same surgical support staff participated in all raTKAs. Planned off-site cadaveric training in raTKA of the three surgeons who participated in the study was not possible due to the lockdowns during the COVID-19 pandemic [13]. Therefore, on-site training was conducted using solid foam anatomical knees (Sawbones, WA, USA).

### 2.2. Surgical Techniques

For both raTKA and maTKA, a traditional surgical incision, a medial parapatellar approach, and the same arthroplasty design (Persona Posterior Stabilized; Zimmer-Biomet, Warsaw, IN, USA) with bone cement (PALACOS R + G Heraeus) were used. Each patient underwent a patellar resurfacing procedure. All surgeries (raTKAs and maTKAs) were performed without limb ischemia and with an uninflated torniquet in place. Unless contraindicated, tranexamic acid was administered intravenously during the surgical procedure and intra-articularly prior to wound closure.

Preoperative planning for maTKA was performed using full-length X-rays of the lower limbs and printed, full-size anteroposterior and lateral X-rays of the knee. Full-length X-rays were used to calculate the femoral and tibial osteotomies; anteroposterior and lateral X-rays of the knee were used to determine the size of the femoral and tibial components of the implant.

In raTKA, manual mapping and landmark acquisition were performed intraoperatively post arthrotomy. Complete limb X-rays and calibrated markers to obtain a 3D virtual model were not performed due to the Spanish Data Protection Act (Organic Law 3/2018).

**Figure 1:**
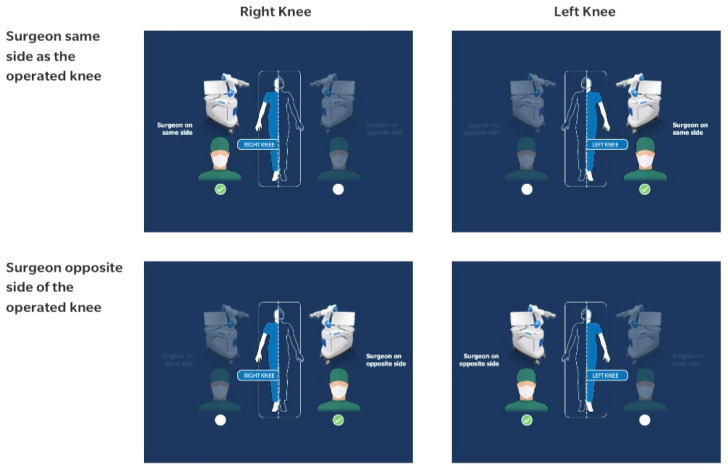
Rosa Knee System surgical technique: options for operating room setup. Used with permission from Zimmer-Biomet, Warsaw, IN, USA.

The surgical technique for raTKA using the ROSA Knee System was performed as recommended by the manufacturer [10,14]. There are several options for the positioning of the robot in the operating room (Figure 1). In our study, the robotic arm was always placed on the same side as the surgeon.

Two surgeons were involved in the procedure: one performed the conventional surgical approach while the other made the skin incisions for the placement of the pins. The femoral pins were placed approximately four fingerbreadths proximal to the skin incision; the tibial pins were placed 4–5 cm distal to the skin incision. Bicortical self-drilling and self-tapping fixed fluted pins (femoral: 3.2 × 150 mm; tibial: 3.2 × 80 mm) were placed through the skin incisions for each NavitrackER. Manual intraoperative mapping was performed with a pointer on the bone surfaces to make anatomical references. Intraoperative dynamic referencing and kinematic evaluation of the knee of the arc of movement from 0° to 45° to 90° was performed with varus–valgus stress, allowing simultaneous testing of the soft tissues. Data were collected in the navigator.

During the intraoperative evaluation, the navigator screen was adjusted as needed. The navigation software (version 1.2) then created and configured the surgical plan. Once the planning was complete, the robotic arm was positioned and held the cutting template at the site where the osteotomy was to be performed. Femoral and tibial osteotomies in the coronal plane were performed perpendicularly to the mechanical axis to achieve neutral alignment. In all raTKAs included in the study, the robot used a predetermined limb axis (mechanical axis) at 0° and a tibial slope at 3°. A freehand saw was then used to cut through the femur and tibia with the help of cutting guides. The surgeon always controlled the saw and guided the cuts. Once the osteotomies were performed, the trial implants were placed to perform an evaluation before the implantation of the definitive components.

In maTKA, conventional instrumentation was based on intramedullary guides for femoral resection (distal cut between 5° and 7° depending on the pre-existing deformity and 3° external rotation using the transepicondylar axis) and for tibial resection perpendicular to the mechanical axis. Ligament balancing was obtained by soft tissue release to equalize the medial and lateral compartments, as well as flexion and extension gaps [11].

### 2.3. Data Collection and Statistical Analysis

The following data were collected for each patient: age, sex, blood hemoglobin and hematocrit (basal and at hospital discharge), and operative time (defined as time in minutes from skin incision to final wound closure). Data were manually entered in Microsoft Excel (Microsoft Corporation, Redmond, WC, USA). Descriptive statistics (frequencies for categorical variables; mean and standard deviation for numerical variables) were used to compare socio-demographic and blood values at baseline. To identify differences across numerical variables between the raTKA and maTKA groups, the Student’s *t*-test or Mann–Whitney test was used. For categorical variables, the Chi-square test or Fischer’s exact test was used.

To estimate the learning curve for raTKA, the cumulative sum control chart method (CUSUM) was used [12]. In this method, values represent a running total of the differences between the value of each data point and the standardized target. A curve was generated using a mixed-regression model; the inflection point represents the transition from the learning phase (the initial increase in the CUSUM until experience is obtained) to the mastered phase (represented by a decline in the CUSUM), which is considered as the post-learning process.

Operative times and blood hemoglobin and hematocrit values in the learning and mastered phases of raTKA were compared with those in maTKA using least squares means and a 95% confidence interval (CI) with a one-way ANOVA test or a non-parametric Kruskal–Wallis test. Pairwise comparisons were applied using the Tukey method. Statistical analyses were performed using STATA 15 (StataCorp LLC, College Station, TX, USA); *p* values < 0.05 were considered significant.

## 3. Results

### 3.1. Participant Characteristics

Characteristics of the study participants are presented in Table 1. There were no differences in demographic characteristics and baseline blood values among the study participants who underwent raTKA or maTKA. Overall, the mean operative time in raTKA was significantly longer compared to that in maTKA (*p* < 0.001).

### 3.2. raTKA Learning Curve

**Figure 2:**
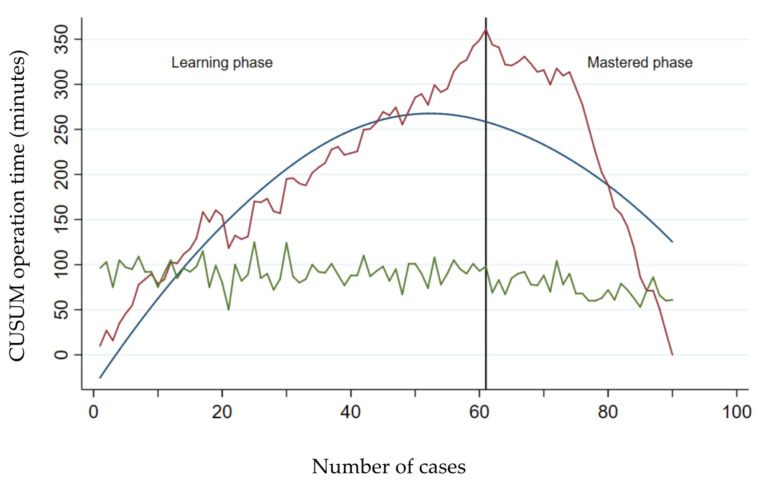
CUSUM analysis of the operative time in raTKA of the three surgeons. Operative times in minutes for maTKA (green curve) and CUSUM of raTKA for the three surgeons combined (red curve) are plotted against the number of surgeries. The vertical line represents the transition from the learning phase to the mastered phase in raTKA. The blue line represents the best fit for the learning curve.

The learning curve for raTKA of the three surgeons combined is shown in Figure 1 and Figure 2. As indicated by the inflection point in the CUSUM curve, the learning phase included 61 cases; the remaining 29 cases fell within the mastered phase.

Operative times in maTKA and in the learning and mastered phases of raTKA of the three surgeons combined are shown in Table 2. In raTKA, the operative times in the learning phase were significantly longer compared to the operative times in the mastered phase (*p* < 0.001). When compared to maTKA, the operative times in the learning and mastered phases of raTKA were also significantly longer (*p* < 0.001). There were no significant differences in the hemoglobin and hematocrit values at hospital discharge between the different groups (maTKA, learning phase of raTKA, and mastered phase of raTKA).

**Figure 3:**
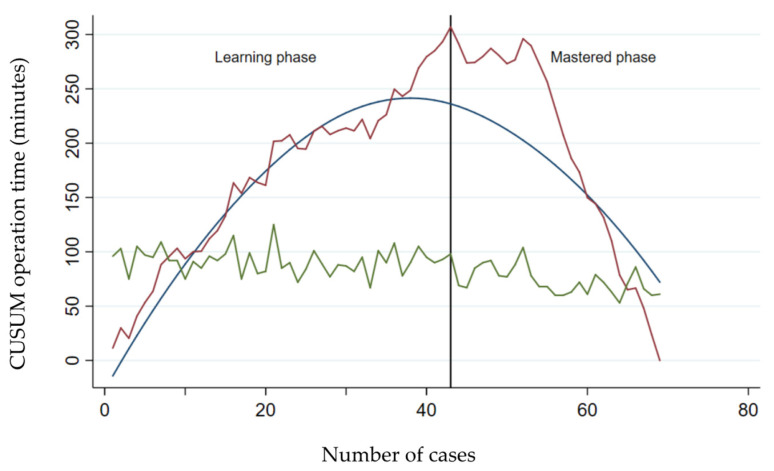
CUSUM analysis of the operative times in raTKA of the experienced surgeon. Operative times in minutes for maTKA (green curve) and CUSUM of raTKA (red curve) are plotted against the number of surgeries. The vertical line represents the transition from the learning phase to the mastered phase in raTKA. The blue line represents the best fit for the learning curve.

To address the possible effect of previous surgical experience in maTKA, the learning curve of surgeon no. 1 (with >15 years of experience in maTKA) was analyzed (Figure 3).

For the 69 raTKAs performed by this surgeon, the learning phase was 43 cases. Similar to the previous analyses, the operative times of the learning phase were significantly longer compared to the mastered phase (*p* < 0.001); in addition, the operative times of the learning and mastered phases in raTKA were significantly longer compared to the operative times in maTKA (*p* < 0.001) (Table 3). However, there were no differences in operative times between the mastered phase in raTKA and maTKA (*p* = 0.087). Also, there were no significant differences in the hemoglobin and hematocrit values at hospital discharge between the raTKA and maTKA groups.

A CUSUM analysis of the operative times in raTKA of the less experienced surgeons alone was not conducted because the experienced surgeon participated in all the surgeries.

## 4. Discussion

In this prospective study, the learning curve for raTKA using the ROSA Knee System was 43 cases for the surgeon with more experience in maTKA and 61 cases for all participating surgeons. The effect of a surgeon´s previous experience in maTKA on the learning curve for raTKA has been previously described [15], and those results are similar to our findings. In addition, the learning curve when adopting a robotic-assisted system for TKA may be affected by the number of cases that are performed using the new technology [7]. In our study, as more cases of raTKA were completed, the surgeons’ confidence with the surgical procedure increased; while one surgeon executed the surgical approach, another surgeon placed the NavitrackERs, which possibly influenced operative times. Finally, although total operative times in raTKA were significantly longer compared to maTKA, blood values at hospital discharge were comparable in the two groups, indicating similar blood losses in both procedures.

Reported learning curves for the ROSA Knee System and for other robotic-assisted systems range from 6 to 43 cases [8,12,15,16,17,18,19,20,21,22,23] (Table S1). The learning curves reported for the ROSA Knee System were 6 to 11 cases in a retrospective study of 90 raTKAs [12], and of 5 to 15 cases in a prospective study of 53 raTKAs [23]. In both studies, all raTKAs were performed by three surgeons with experience in maTKA, with the short learning curves suggesting that the ROSA Knee System could be incorporated relatively quickly by surgical teams. The reasons for the longer learning curve observed in our study using the ROSA Knee System are probably multifactorial, including the study’s prospective nature, a difference in the previous experience of the participating surgeons in maTKA, an absence of prior cadaveric training in raTKA, and the disruptions caused by the periodic cancellations of elective surgeries due to COVID-19 lockdowns during the study period [13]. In addition, only three additional centers across our country were starting to use the ROSA Knee System at the time of our study, which further limited our learning. It is likely that if the study were conducted today, the learning curve would probably be shorter. Nonetheless, our results represent the real-life experience of incorporating raTKA in a public institution whose surgeons lacked prior experience in robotic-assisted surgery.

The MAKO Robotic Arm Interactive Orthopedic System (Stryker Ltd., Kalamazoo, MI, USA) is an image-guided semiactive robot system that requires a preoperative CT scan to generate a 3D model of the patient´s bone anatomy [9]. The published learning curves for this system range from 7 to 43 cases [15,16,17,21,22]. In a large study of 240 raTKAs performed by two experienced surgeons, a learning curve of 20 cases was based on the mean operative times of raTKAs grouped into 20 sequential cases for each surgeon [16]. In one retrospective study of 60 raTKAs performed by a single surgeon, the reported learning curve for operative time and surgical team comfort levels was 7 cases [17]. In a prospective series of 31 raTKAs performed by three surgeons with different surgical experience, the learning curve for operative times was 9 cases; the authors of that study concluded that the participation of a surgeon with previous experience in raTKA may flatten the learning curve of a surgical team without experience in robotic-assisted systems [15]. In a large retrospective study involving 386 raTKAs and six participating surgeons, the learning curve ranged from 11 to 43 cases; the authors suggested that the longer learning curve could be dependent on the profile of the surgeon [21]. In another retrospective study of 120 raTKAs performed by two surgeons, a similar time frame to maTKA was achieved within the first 40 raTKAs [22]. Using this system, a prospective study has shown that the initial learning curve for a single surgeon continues to decrease after six months and one year [24]. It has also been noted that the integration of the MAKO Robotic Arm Interactive Orthopedic System in the operating room can be achieved with the willingness and participation of all members of the surgical team [25].

The TSolution One (THINK Surgical, Freemont, CA, USA) is an image-based autonomous milling robotic system [9]. A prospective study that analyzed 107 raTKAs performed by four experienced surgeons using this system showed variable learning curves for each surgeon that ranged from 10 to 20 cases [19].

The NAVIO Surgical System (Smith & Nephew, Andover, TX, USA) is an imageless robotic system equipped with a handheld end-cutting burr that provides live intraoperative feedback [9]. The published learning curves for this system range from 7 to 29 cases [8,18,20]. In a prospective study of 75 raTKAs performed by a single surgeon, there was a short learning curve of 7 cases [18]. In another prospective study of 70 raTKAs also performed by a single surgeon, the learning curve was completed after 11 cases [8]. Finally, in a prospective study of 60 raTKAs, a single senior surgeon completed the learning curve after 29 cases but reported a high level of confidence with the system after 10 cases [20]. Consistent with our findings, the reported learning curves of the different robotic-assisted systems for TKA described above suggest that prior experience in maTKA significantly influences the duration of the learning curve for raTKA.

Incorporating raTKA into a center whose surgeons lack previous experience in robotic surgery would require specific training of orthopedic surgeons through cadaveric courses or fellowships in centers whose surgeons have established experience with those systems. As supported by our results and those from other studies, it is desirable for the surgeons receiving training in raTKA to have prior experience in maTKA to shorten the learning curve regarding their training on the new system. In addition, the willingness of the entire surgical team is crucial for the successful adoption of raTKA. Because the learning curve is affected by the number of cases performed, surgical centers considering the implementation of raTKA should assume that it would take some time before operative times equal those of maTKA.

Our study was not designed to evaluate outcomes following raTKA. Due to the relatively recent adoption of robotic-assisted systems for TKA, there is a paucity of long-term follow-up studies of patients undergoing raTKA. As recently noted, there is a need for clinical trials with well-defined endpoints to determine whether raTKA improves long-term outcomes and patient satisfaction compared to maTKA [26].

There are several limitations to our study: (1) our patients were chosen sequentially and were not randomized; (2) preoperative planning based on imaging was limited due to local regulations; (3) previous experience of the participating surgeons in maTKA varied; (4) our findings might not be applicable to other surgical centers with different patient populations and surgeon experience in maTKA; and (5) the reduced enrollment of patients in the study due to lockdowns during the COVID-19 pandemic, which possibly slowed down the learning curve of the surgeons who were trained in raTKA.

## 5. Conclusions

In this prospective study conducted at a single surgical center whose surgeons lacked previous experience in raTKA, the learning curve for the ROSA Knee System was 43 and 61 cases. The operative times in the learning and mastered phases of raTKA were significantly longer compared to those of maTKA. The operative times in the learning phase were significantly longer compared to those in the mastered phase in raTKA; however, there were no differences in operative times of the experienced surgeon in the mastered phase of raTKA and in maTKA. The number of cases needed to reach the inflection point in the learning curve of raTKA is dependent upon the surgeon’s experience in maTKA. The results of our study could be helpful to other surgical centers planning to adopt robotic-assisted systems for TKA.

## Figures and Tables

**Table 1:** Baseline characteristics of study participants.

	All (N = 180)	raTKA (N = 90)	maTKA (N = 90)	*p*-Value ^1^
Demographics				
Age [years (SD)]	72.4 (7.7)	72.5 (7.1)	72.3 (8.4)	0.945
Gender [N (%)]				
Female	120 (68)	59 (66)	61 (68)	0.752
Male	60 (33)	31 (34)	29 (32)	0.752
Operative time [minutes (SD)]	76.8 (17.2)	86.0 (15.3)	67.5 (13.7)	<0.001
Blood values				
Hematocrit [% (SD)]	44 (3.4)	43 (3.4)	44 (3.5)	0.230
Hemoglobin [g/dL (SD)]	14 (1.1)	14 (1.2)	14 (1.1)	0.912

^1^ Student’s *t*-test, Mann–Whitney test, or Chi-square test.

**Table 2:** Comparison of learning and mastered phases in raTKA with operative times in maTKA of the three surgeons combined.

	maTKA		Learning Phase raTKA		Mastered Phase raTKA		
Operative Time	LSM (SE)	95% CI	LSM (SE)	95% CI	LSM (SE)	95% CI	*p*-Value ^1^
Total minutes	67.5 (1.4)	64.7–70.3	91.9 (1.7)	88.4–95.3	74.4 (2.4)	69.6–79.2	<0.001 ^2^
Blood values at hospital discharge							
Hematocrit [%]	33.4 (0.4)	32.6–31.2	34.3 (0.5)	33.4–35.3	32.7 (0.7)	31.3–34.1	0.121
Hemoglobin [g/dL]	11.0 (0.1)	10.7–11.2	11.3 (0.2)	11.0–11.6	10.9 (0.2)	10.4–11.3	0.216

Abbreviations: maTKA, conventional TKA; raTKA, robotic-assisted TKA; LSM, least squares mean; SE, standard deviation; CI, confidence interval. ^1^ One-way ANOVA and Kruskal–Wallis tests. ^2^ Pairwise comparisons were adjusted with the Tukey method. Differences in operative times of the learning phase in raTKA and maTKA (mean difference: 24.3; 95% CI: 19.1–29.6), operative times of the mastered phase in raTKA and maTKA (mean difference: 6.9; 95% CI: 0.2–13.5), and operative times of the mastered and learning phases in raTKA (mean difference: −17.5; 95% CI: −24.5–−10.4)) were statistically significant (*p*-values < 0.001, <0.041, and <0.001, respectively).

**Table 3:** Comparison of learning and mastered phases in raTKA with operative times in maTKA of the experienced surgeon.

	maTKA		Learning Phase raTKA		Mastered Phase raTKA		
Operative Time	LSM (SE)	95% CI	LSM (SE)	95% CI	LSM (SE)	95% CI	*p*-Value ^1^
Total minutes	67.5 (1.4)	64.7–70.3	91.5 (2.0)	87.5–95.5	73.7 (2.5)	68.7–78.7	<0.001 ^2^ 0.087 ^2,3^
Blood values at hospital discharge							
Hematocrit [%]	33.4 (0.4)	32.6–31.2	34.2 (0.6)	33.0–35.4	32.7 (0.7)	31.3–34.2	0.279
Hemoglobin [g/dL]	11.0 (0.1)	10.7–11.2	11.2 (0.2)	10.8–11.6	10.9 (0.2)	10.4–11.4	0.485

Abbreviations: maTKA, conventional TKA; raTKA, robotic-assisted TKA; LSM, least squares mean; SE, standard deviation; CI, confidence interval. ^1^ One-way ANOVA and Kruskal–Wallis tests. ^2^ Pairwise comparisons were adjusted with the Tukey method. Differences in operative times of the learning phase in raTKA and maTKA and differences in operative times of the learning and mastered phases in raTKA were statistically significant (mean difference: −17.9; 95% CI: −25.5–−10.2; *p*-value < 0.001). ^3^ Differences in operative times of the mastered phase in raTKA and maTKA were not statistically significant (mean difference: 6.1; 95% CI: −0.7–13.0; *p*-value = 0.087).

## Data Availability

The data presented in this study are available on request from the corresponding author.

## Supplementary Materials

The following supporting information can be downloaded at: https://www.mdpi.com/article/10.3390/jcm13113349/s1; Table S1. Learning curves of different robotic systems for TKA.

## References

[1] St. Mart J.-P., Goh E.L. (2021). The current state of robotics in total knee arthroplasty. EFORT Open Rev..

[2] Bourne R., Chesworth B., Davis A., Mahomed N., Charron K. (2018). Patient satisfaction after total knee replacement: A systematic review. HSS J..

[3] Halawi M.J., Jongbloed W., Baron S., Savoy L., Williams V.J., Cote M.P. (2019). Patient dissatisfaction after primary total joint arthroplasty: The patient perspective. J. Arthroplast..

[4] Parrate S., Price A.J., Jeys L.M., Jackson W.F., Clarke H.D. (2019). Accuracy of a new robotically assisted technique for total knee arthroplasty: A cadaveric study. J. Arthroplast..

[5] Kayani B., Haddad F.S. (2019). Robotic total knee arthroplasty: Clinical outcomes and directions for future research. Bone Jt. Res..

[6] Kayani B., Konan S., Ayuob A., Onochie E., Al-Jabri T., Haddad F.S. (2019). Robotic technology in total knee arthroplasty: A systematic review. EFORT Open Rev..

[7] Polikandriotis J.A., Cafferky N.L. (2021). Question and answer—Integrating a robotic assistant into a high-volume orthopedic practice. J. Orthop. Exp. Innov..

[8] Savov P., Tuecking L.-R., Windhagen H., Ehmig J., Ettinger M. (2021). Imageless robotic handpiece-assisted total knee arthroplasty: A learning curve analysis of surgical time and alignment accuracy. Arch. Orthop. Trauma. Surg..

[9] Mancino F., Jones C., Benazzo F., Singlitico A., Giuliani A., De Martino I. (2022). Where are we now and what are we hoping to achieve with robotic total knee arthroplasty? A critical analysis of the current knowledge and future perspectives. Orthop. Res. Rev..

[10] Klein G., James D., Lonner J., Lonner J.H. (2019). Total knee arthroplasty technique: ROSA knee. Robotics in Knee and Hip Arthroplasty.

[11] Batailler C., Hannouche D., Benazzo F., Parratte S. (2021). Concepts and techniques of a new robotically assisted technique for total knee arthroplasty: The ROSA knee system. Arch. Orthop. Trauma. Surg..

[12] Vanlommel L., Neven E., Anderson M.B., Bruckers L., Truijen J. (2021). The initial learning curve for the ROSA^®^ Knee System can be achieved in 6–11 cases for operative time and has similar 90-day complication rates with improved implant alignment compared to manual instrumentation in total knee arthroplasty. J. Exp. Orthop..

[13] Información Microbiológica Acerca del SARS-CoV-2. https://www.sanidad.gob.es/areas/alertasEmergenciasSanitarias/alertasActuales/nCov/documentos/20220113_MICROBIOLOGIA.

[14] (2023). ROSA Knee.

[15] Schopper C., Proier P., Luger M., Gotterbarm T., Klasan A. (2023). The learning curve in robotic assisted knee arthroplasty is flattened by the presence of a surgeon experienced with robotic assisted surgery. Knee Surg. Sports Traumatol. Arthrosc..

[16] Sodhi N., Khlopas A., Piuzzi N.S., Sultan A.A., Marchand R.C., Malkani A.L., Mont M.A. (2018). The learning curve associated with robotic total knee arthroplasty. J. Knee Surg..

[17] Kayani B., Konan S., Huq S.S., Tahmassebi J., Haddad F.S. (2019). Robotic-arm assisted total knee arthroplasty has a learning curve of seven cases for integration into the surgical workflow but no learning curve effect for accuracy of implant positioning. Knee Surg. Sports Traumatol. Arthrosc..

[18] Thiengwittayaporn S., Uthaitas P., Senwiruch C., Hongku N., Tunyasuwanakul R. (2021). Imageless robotic-assisted total knee arthroplasty accurately restores the radiological alignment with a short learning curve: A randomized controlled trial. Int. Orthop..

[19] Mahure S.A., Teo G.M., Kissin Y.D., Stulberg B.N., Kreuzer S., Long W.J. (2022). Learning curve for active robotic total knee arthroplasty. Knee Surg. Sports Traumatol. Arthrosc..

[20] Bell C., Grau L., Orozco F., Ponzio D., Post Z., Czymek M., Ong A. (2022). The successful implementation of the Navio robotic technology required 29 cases. J. Robot. Surg..

[21] Vermue H., Luyckx T., Winnock de Grave P., Ryckaert A., Cools A.S., Himpe N., Victor J. (2022). Robot-assisted total knee arthroplasty is associated with a learning curve for surgical time but not for component alignment, limb alignment and gap balancing. Knee Surg. Sports Traumatol. Arthrosc..

[22] Ali M., Phillips D., Kamson A., Nivar I., Dahl R., Hallock R. (2022). Learning curve of robotic-assisted total knee arthroplasty for non-fellowship-trained orthopedic surgeons. Arthroplast. Today.

[23] Bolam S.M., Tay M.L., Zaidi F., Sidaginamale R.P., Hanlon M., Munro J.T., Monk P. (2022). Introduction of ROSA robotic-arm system for total knee arthroplasty is associated with a minimal learning curve for operative time. J. Exp. Othop.

[24] Marchand K.B., Ehiorobo J., Mathew K.K., Marchand R.C., Mont M.A. (2022). Learning curve of robotic-assisted total knee arthroplasty for a high-volume surgeon. J. Knee Surg..

[25] Coon T.M. (2009). Integrating robotic technology in the operating room. Am. J. Orthop..

[26] Khatri C., Netcalfe A., Wall P., Underwood M., Haddad F.S., Davis E.T. (2024). Robotic trials in arthroplasty surgery. Des. RACER Stud. Implic. Future. Bone Jt. J..

