# Supplementary material for: Analysis of the Initial Learning Curve for Robotic-Assisted Total Knee Arthroplasty Using the ROSA® Knee System
Source: J Clin Med. 2024 Jun 6;13(11):3349. doi: 10.3390/jcm13113349 (PMC11172834; doi:10.3390/jcm13113349)
Supplement: Supplementary file 1 [file jcm-13-03349-s001.zip › jcm-2987589-supplementary.pdf]

Supplementary Materials

A review of articles was performed in MEDLINE, PubMed and Google Scholar using the following terms: total knee arthroplasty (TKA), robotics, robotic surgery, robotic total knee arthroplasty, learning curve, and ROSA knee system. Search was limited to articles published in the last 10 years, involving human subjects, and published in English language. Published studies on raTKA that include specific data on the learning curve are summarized in Table S1.

Table S1. Learning curves of different robotic systems for TKA

**Author [reference]**	**Year**	**Robotic system**	**Manufacturer**	**Number of raTKA analyzed**	**Learning curve (cases)**
Sodhi [16]	2018	MAKO	Stryker Ltd, Kalamazoo, MI, USA	240	20
Kayani [17]	2019	MAKO	Stryker Ltd, Kalamazoo, MI, USA	60	7
Vanlommel [12]	2021	ROSA	Zimmer-Biomet, Warsaw, IN, USA	90	6-11
Thiengwittayaporn [18]	2021	NAVIO	Smith & Nephew, Andover, TX, USA	75	7
Savov [8]	2021	NAVIO	Smith & Nephew, Andover, TX, USA	70	11
Mahure [19]	2022	TSolution-One	THINK Surgical, Freemont, CA, USA	107	10-20
Bell [20]	2022	NAVIO	Smith & Nephew, Andover, TX, USA	60	29
Schopper [15]	2022	MAKO	Stryker Ltd, Kalamazoo, MI, USA	31	9
Vermue [21]	2022	MAKO	Stryker Ltd, Kalamazoo, MI, USA	386	11-43
Ali [22]	2022	MAKO	Stryker Ltd, Kalamazoo, MI, USA	120	40
Bolam [23]	2022	ROSA	Zimmer-Biomet, Warsaw, IN, USA	53	5-15
